# Honokiol, a Neolignan from *Magnolia officinalis*, Attenuated Fructose-Induced Hepatic Fat Accumulation by Improving Intestinal Barrier Function in Mice

**DOI:** 10.1016/j.tjnut.2025.02.017

**Published:** 2025-02-21

**Authors:** Anja Baumann, Verena Freutsmiedl, Julia Jelleschitz, Raphaela Staltner, Annette Brandt, Daniel Schachner, Verena M Dirsch, Ina Bergheim

**Affiliations:** 1 Department of Nutritional Sciences, Molecular Nutritional Science, University of Vienna, Vienna, Austria; 2 Department of Pharmaceutical Sciences, University of Vienna, Vienna, Austria

**Keywords:** neolignans, fructose, intestinal barrier dysfunction, NO homeostasis, MASLD

## Abstract

**Background:**

Fructose (Fru) consumption has been suggested to contribute to metabolic diseases including metabolic dysfunction-associated steatotic liver disease (MASLD), at least in part, by disturbing intestinal barrier function and intestinal nitric oxide (NO) homeostasis. Honokiol (Hon), a neolignan found in *Magnolia officinalis*, has been suggested to affect intestinal integrity and barrier function.

**Objectives:**

We assessed whether Hon affects Fru-induced small intestinal permeability in settings of early MASLD.

**Methods:**

Female 8–10-wk-old C57BL/6J mice (*n =* 7/group) received either a 30% Fru solution + vehicle or plain drinking water + vehicle ± Hon (10 mg/kg bw/d) for 4 wk. Liver damage [e.g. nonalcoholic fatty liver disease activity score (NAS), number of neutrophils, interleukin-6 (IL-6) protein concentration], markers of intestinal permeability (bacterial endotoxin, tight junction proteins), and NO homeostasis in the small intestine were determined in vivo as well as ex vivo in an everted sac model and in Caco-2 cells. One-way and 2-way analysis of variance were performed, respectively.

**Results:**

Hon diminished the development of MASLD, which was associated with a significant lower NAS (–38%), number of neutrophils (–48%), and IL-6 protein concentrations (–38%) in livers of Fru-fed mice. Hon also attenuated Fru-induced alterations of markers of intestinal barrier function with Fru+Hon-fed mice showing lower bacterial toxin levels in portal plasma (–29%, *P* = 0.075), higher tight junction protein concentrations (+2.4-fold, *P* < 0.05), and lower NOx concentration (–44%, *P* < 0.05) as well as NO synthase activity (–35%) in the small intestine compared with Fru+vehicle-fed mice. Moreover, the decrease in AMP-activated protein kinase phosphorylation found in the small intestine of Fru-fed mice was significantly attenuated (+5.3-fold) by the concomitant treatment with Hon in Fru-fed mice. In support of the in vivo findings, Hon significantly attenuated Fru-induced intestinal permeability ex vivo and in Caco-2 cells.

**Conclusions:**

Our data suggest that Hon diminished the development of Fru-induced early MASLD by alleviating impairments in intestinal barrier function.

## Introduction

Metabolic dysfunction-associated steatotic liver disease (MASLD), which encompasses a broad spectrum of disease, ranging from simple steatosis to metabolic dysfunction-associated steatohepatitis (MASH) to fibrosis and even cirrhosis, is by now the most prevalent liver disease worldwide [1]. Indeed, it is estimated that with still increasing numbers ∼32% of the world’s general population is now affected [2,3].

During the last decade, several factors have been identified that increase the odds to develop MASLD, among which general overnutrition but also diet composition seem to be key factors. Indeed, diets rich in free fructose (Fru) are discussed to contribute to MASLD development (for overview, see [4]). Results from animal models but also from some human studies have suggested that an intake of elevated amounts of free Fru from sweetened drinks or foods may increase hepatic fat content [[5], [6], [7]] being associated with impairments of intestinal barrier function and elevated bacterial endotoxin levels in the portal and peripheral blood [8,9]. By binding to Toll-like receptor 4 (TLR4) and activating downstream signaling cascades, bacterial endotoxin can induce inflammatory responses in liver tissue and modulate hepatic lipid metabolism [10,11]. Indeed, in recent years, several signaling cascades have been identified that are critical in the development of MASLD and the first drug for the treatment of MASH with fibrosis has been approved [12]. However, due to the lack of fully understanding underlying mechanisms, universally accepted therapies of MASLD that go beyond lifestyle interventions, such as focusing on a reduction of body weight and an increase in physical activity, are still lacking.

Honokiol (Hon) is a neolignan found in the bark, seed cones, and leaves of several *Magnolia* species. Magnolia bark has been used for centuries in traditional Chinese and Japanese medicine as herbal preparation [13]. Nowadays, extracts of Magnolia bark are used in many dietary supplements and food additives as well as teas worldwide (for overview see [14]). Hon (and magnolol) contents in commercially available Magnolia bark extracts range from 40% to 90% of total polyphenols, depending on plant species, area of origin, and preparation of the extract (for overview, see [14]). On the basis of the results of toxicological studies in rats, a no-observed-adverse-effect-level for concentrated Magnolia bark extracts was established to be >240 mg/kg bw/d for oral consumption [15]. Hon has been shown to have a wide range of therapeutic effects including antidepressant, antitumor, and analgesic properties [16,17]. Recently, it was shown in a mouse model of high-fat diet-induced MASLD that Hon can attenuate the development of insulin resistance and MASLD through mechanisms involving an inhibition of AMP-activated protein kinase (AMPK)γ1 in hepatocytes, whereas in another study employing a model of methionine choline-deficient (MCD) diet-induced MASLD protective effects found on the development of MASLD seem not to be primarily related to its effects on intestinal microbiota composition and bile acid metabolism [18]. Moreover, in the setting of dextran sulfate sodium (DSS)-induced colitis, Hon has been suggested to improve intestinal barrier function through reducing markers of inflammation, e.g. expression of *IL-1β*, *IL-6,* and *TNF α* mRNA, inhibiting oxidative stress and recovering tight junction proteins and mucins in the colon. These protective effects were related to the interference with AMPK/nuclear factor erythroid 2-related factor 2/heme oxygenase 1 antioxidant pathways and sirtuin 3/AMPK energy regulation pathways [19]. However, whether Hon also has protective effects on intestinal barrier function in the setting of Fru-induced MASLD, and thereby affecting the development of MASLD, remains to be determined.

Starting from this background, this study aimed to determine whether a concomitant oral treatment of mice with Hon can attenuate the development of Fru-induced fat accumulation in liver tissue and whether this is related to alterations of intestinal barrier function.

## Methods

### Animals and treatment

A total of 8–10-wk-old female C57BL/6J mice (Janvier SAS) were housed in a specific pathogen-free facility accredited by the Association for Assessment and Accreditation of Laboratory Animal Care. All procedures were approved by the local Institutional Animal Care and Use Committee (Federal Ministry Republic of Austria Education, Science and Research, Vienna, Austria, 2021-0.785.831). Mice (*n =* 7/group, in total 4 experimental groups) had free access to standard feed pellets (catalog number V1534-300, pellets, fortified, Ssniff; for diet composition, see Supplemental Table 1). Mice were randomly assigned to the following experimental groups and treated for 4 wk: Control (C), mice receiving tap water + vehicle; C+Hon, mice receiving tap water enriched with 10 mg Hon/kg body weight/d; Fru, mice receiving 30% (w/v) Fru solution + vehicle; Fru+Hon, mice receiving 30% (w/v) Fru solution + 10 mg Hon/kg body weight/d. Because Hon (Glentham Life Sciences) is not soluble in water, Hon was dissolved in DMSO before adding to drinking solutions. The final concentration of DMSO (vehicle) was 0.12% in all experimental groups. The concentration of Hon was based on previous studies of others [20]. The sample size was determined based on previous findings [21]. Body weight was assessed weekly, the consumption of feed pellets was assessed twice weekly and drinking solution was assessed daily. Fasting blood glucose levels were determined after an overnight fast 1 wk before the end of the experiment. At the end of the experiment, mice were terminal anesthetized with 100 mg ketamine and 16 mg xylazine/kg body weight and killed by cervical dislocation. Blood was collected from the portal vein. The liver and small intestine were collected and fixed in neutral-buffered formalin or snap-frozen for further analyses. To determine small intestinal permeability ex vivo, parts of the small intestine were everted with a rod as previously described [22,23]. Small intestinal tissue sacs were ligated at both ends and filled with 1× Krebs–Henseleit-bicarbonate-buffer supplemented with 0.2% bovine serum albumin (KRH buffer). To determine intestinal permeability, permeation of xylose was measured by incubating everted gut sacs in a 0.1% D-xylose solution for 5 min in gassed conditions. After incubation, liquids inside the everted gut sacs were collected as well as intestinal tissue was snap-frozen for further analyses. Moreover, enterocytes were isolated from the proximal small intestine as detailed by Cartwright and Higgins [24].

### Everted gut sac experiment and determination of small intestinal permeability ex vivo

**FIGURE 1:**
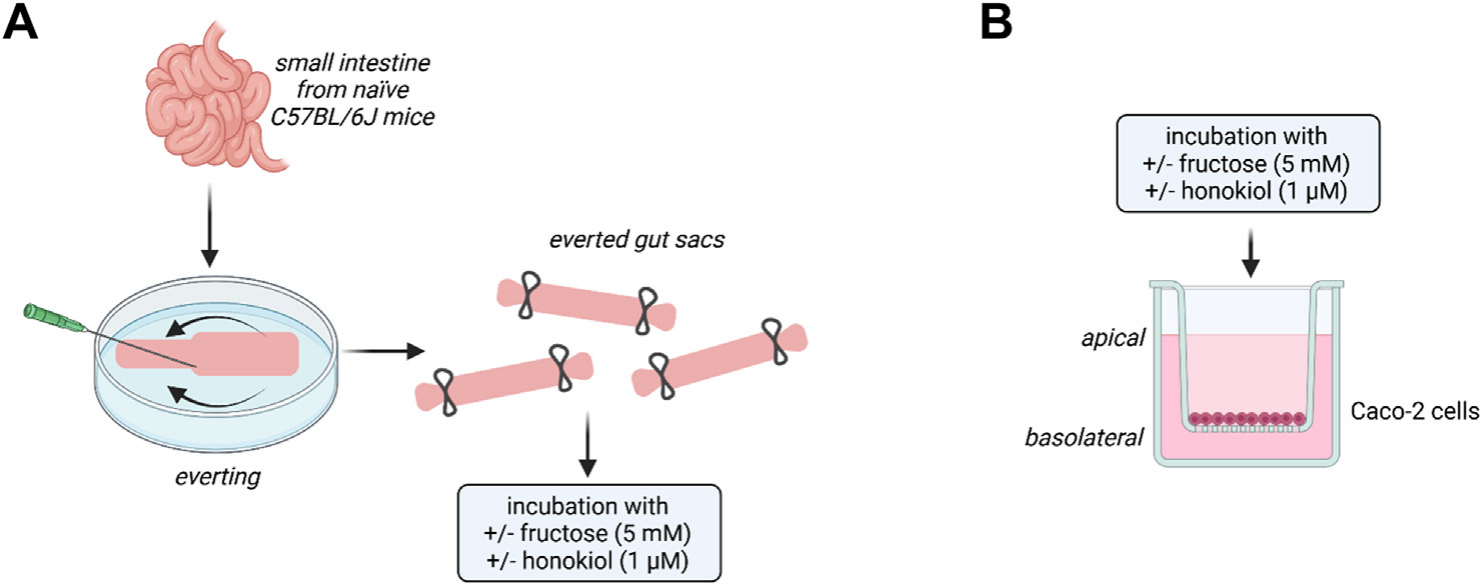
Schematic drawing of the experimental setup of (A) ex vivo everted sac experiments and (B) Caco-2 cell experiment. Figures were created with BioRender.com.

Small intestinal tissue from naïve female C57BL/6J mice (Janvier Labs) was collected and rinsed with phosphate-buffered saline (PBS). Tissue was cut into pieces and everted gut sacs were prepared as detailed above. Everted gut tissue sacs were preincubated in 1× KRH buffer ± 1 μM Hon for 10 min, followed by an incubation ±5 mM Fru and ±1 μM Hon for 55 min at 37°C in a gassed 95% oxygen/5% carbon dioxide atmosphere. The study design is shown in Figure 1A. To determine intestinal permeability, permeation of xylose was measured by incubating everted gut sacs in a 0.1% D-xylose solution for 5 min.

### Caco-2 cell experiments and determination of permeability

Caco-2 cells were obtained from the German Collection of Microorganisms and Cell Culture (Leibniz Institute DSMZ-German Collection of Microorganisms and Cell Culture GmbH) and maintained in Eagle's minimum essential medium (Sigma Aldrich Handels GmbH) supplemented with 20% heat-inactivated fetal bovine serum (Biowest, VWR International), 2 mM L-glutamine (Sigma Aldrich Handels GmbH), 100 U/mL penicillin, 100 μg/mL streptomycin (Sigma Aldrich Handels GmbH), and 1× nonessential amino acids (Sigma Aldrich Handels GmbH) at 37°C in an incubator with 5% carbon dioxide. The cultivation and differentiation protocol is described in more detail in Hiebl et al. [25]. The permeability of the epithelial barrier of Caco-2 cells was evaluated with DextranBlue as detailed in [26] with slight modifications [25]. Differentiated Caco-2 cells were apically treated with 1 μM Hon or solvent control (Sigma Aldrich Handels GmbH) for 10 min. After preincubation, 5 mM Fru and 20 mg/mL DextranBlue (Sigma Aldrich Handels GmbH) were added for another 30 min. The study design is shown in Figure 1B. A total of 100 μL of the basolateral solution (no human plasma as acceptor) was quantified using a Tecan Spark plate reader at 620 nm.

### Evaluation of liver damage

Sections of paraffin-embedded liver tissue (4 μm) were prepared and stained with hematoxylin and eosin (Sigma Aldrich GmbH). Liver damage (steatosis grade, lobular inflammation, and ballooning of hepatocytes) was evaluated by employing a scoring system referred to as the nonalcoholic fatty liver disease activity score (NAS) modified from Kleiner et al. [27], and Brunt [28]. Neutrophils were stained with naphthol AS-D chloroacetate specific Esterase Kit (Sigma Aldrich Handels GmbH) and the number of neutrophils were counted per microscopic field. For determination of hepatic fat accumulation, frozen liver sections (10 μm) were stained with Oil Red O (Sigma Aldrich GmbH) for 12 min, washed and counterstained with hematoxylin (Sigma Aldrich GmbH) for 20 s as detailed before [29]. Representative pictures were taken using a microscope with an integrated camera (Leica DM6B, Leica DMC4500. Leica).

### Endotoxin assay and measurement of TLR2 ligands

Bacterial endotoxin levels in plasma were measured with a commercially available Limulus amebocyte lysate assay (Charles River) as described in detail before [30]. Ligands of TLR2 in portal plasma were measured with HEK-Blue-mTLR2 cells (InvivoGen) obtained by cotransfection of the murine TLR2 and secretion of embryonic alkaline phosphatase reporter genes into HEK293 cells as detailed previously [31].

### Nitrite (NO_2_^–^ or NO_X_), NO synthase (NOS), and arginase activity assay

NO_2_^–^ levels in intestinal tissue were measured with a commercially available kit (Promega Corporation). Activity of NOS in enterocytes was measured with a fluorometric NOS activity assay kit (Abcam). To measure arginase activity in total small intestine and in enterocytes, tissue, and cells were homogenized in 10 mM Tris–HCl containing 0.4% (w/v) triton X-100 and protease inhibitor cocktail and the assay was performed as detailed previously [32].

### Plasminogen activator inhibitor-1 (PAI-1), myeloperoxidase (MPO) activity, and thiobarbituric acid reactive substances (TBARS) concentration

To determine total PAI-1, liver tissue was homogenized in PBS and the concentration of PAI-1 was measured with a commercially available kit (LOXO GmbH). MPO, mainly released by neutrophils [33], was measured in liver tissue as detailed before [34]. To assess TBARS, a byproduct of lipid oxidation [35], liver tissue was lysed in radioimmunoprecipitation assay buffer (RIPA buffer) containing protease inhibitors. After precipitation with 10% trichloroacetic acid, the supernatant was incubated with thiobarbituric acid for 10 min at 95°C. The concentration of TBARS was measured at 532 nm with a photometer (SpectraMax, Molecular Devices).

### Immunohistochemical staining of the tight junction protein zona occludens-1 (ZO-1) in small intestinal tissue

Paraffin-embedded small intestinal tissue sections (4 μm) were stained with ZO-1 as detailed before [36]. In brief, sections were incubated with the primary antibody for ZO-1 (Thermo Fisher Scientific), followed by incubation with a peroxidase-linked secondary antibody and 3,3′-diaminobenzidine chromogen solution staining (Agilent Dako). Representative images were captured using a microscope (Leica DM6 B, Leica).

### Western blot analysis

To obtain total protein, small intestinal tissue was lysed in TRItidy G (PanReac AppliChem, VWR International) according to the manufacturer's instructions. Protein lysates were separated in a sodium dodecylsulfate polyacrylamide gel electrophoresis and transferred on a polyvinylidene difluoride membrane. Membranes were then incubated with the primary antibodies against occludin (Thermo Fisher Scientific), phospho-AMPK alpha (AMPKα, Thr172; Cell Signaling), AMPK alpha 1 and 2 (Abcam), or β-actin (Santa Cruz Biotechnology) followed by incubation with secondary antibodies (anti-mouse or anti-rabbit, Cell Signaling), respectively. Bands were visualized with Clarity Western Enhanced Chemiluminescence Substrate (Bio-Rad Laboratories). Densitometric analyses of detected bands were performed with ImageLab (Bio-Rad Laboratories).

### Statistical analysis

Data are shown as means ± SEMs. Outliers were identified using Grubb’s test. Statistical analyses were performed with GraphPad Prism 7.0 software (GraphPad Prism Software). Data were log-transformed when they were not normally distributed or in case of inhomogeneity of variances. One-way and 2-way analysis of variance was performed to determine differences between different treatment groups followed by Tukey’s post hoc test. A *P* ≤0.05 was defined to be statistically different.

## Results

### Effect of Hon on markers of liver damage and inflammation in mice with Fru-induced MASLD

**FIGURE 2:**
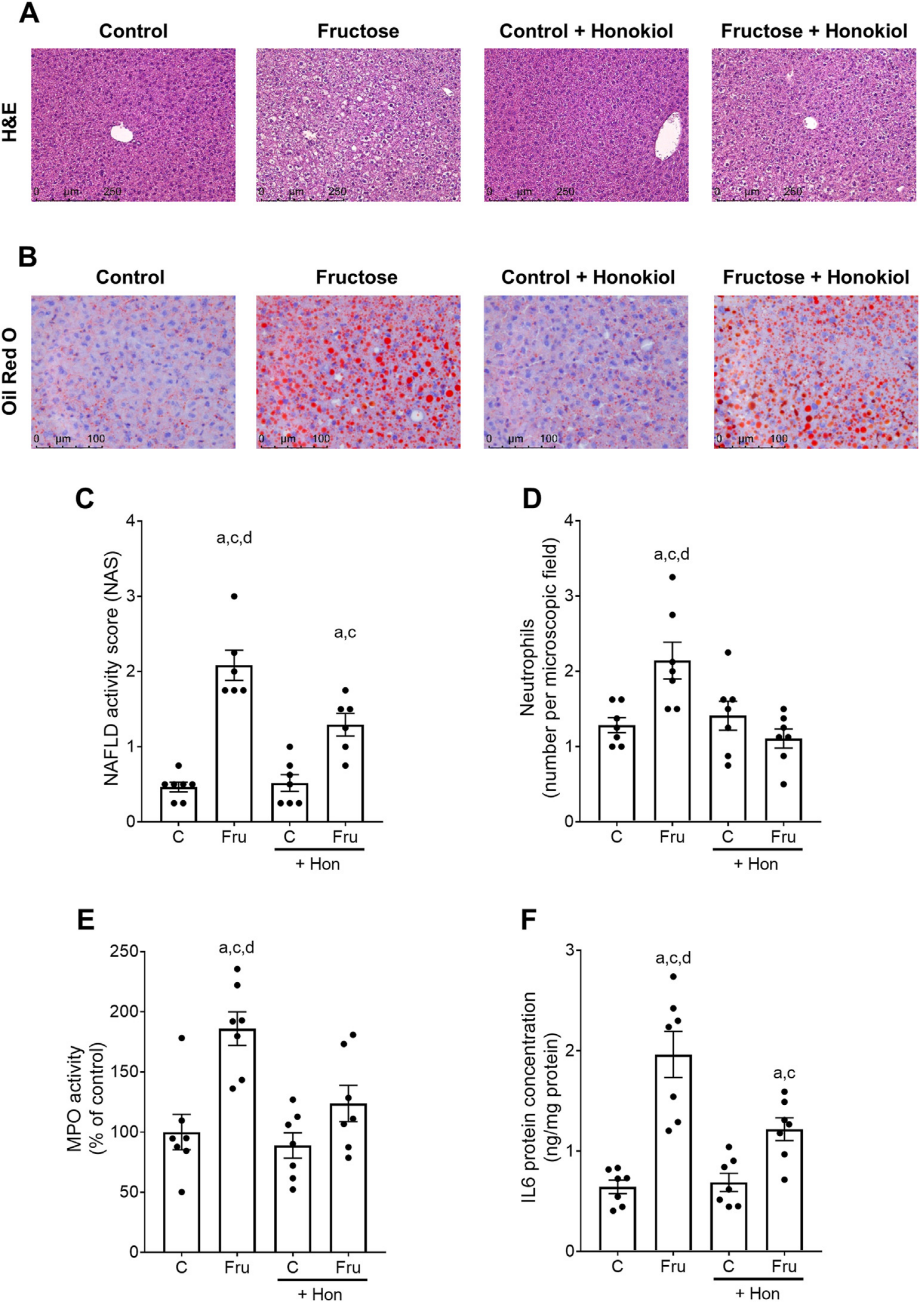
Effect of honokiol on markers of liver damage in mice with fructose-induced MASLD. Representative pictures of (A) hematoxylin and eosin staining (H&E, magnification 200×) and (B) Oil Red O staining (magnification 400×) in liver tissue, (C) NAFLD activity score (NAS), (D) number of neutrophils, (E) activity of myeloperoxidase (MPO) and (F) IL-6 protein concentration in livers. Data are presented as means ± SEM, *n* = 6–7, ^a^*P* ≤ 0.05 compared with mice fed a control diet, ^c^*P* ≤ 0.05 compared with mice fed a control diet and treated with 10 mg honokiol/kg bw, ^d^*P* ≤ 0.05 compared with mice fed a 30% fructose solution and treated with 10 mg honokiol/kg bw. C, control diet; Fru, 30% fructose solution; Hon, honokiol; MASLD, metabolic dysfunction-associated steatotic liver disease; NAFLD, nonalcoholic fatty liver disease.

As expected and in line with previous findings [21] and despite no differences in body weight, Fru-fed mice had developed steatosis with beginning signs of hepatic inflammation after 4 wk of feeding. These alterations were significantly attenuated in livers of Fru-fed mice concomitantly treated with Hon. Specifically, NAS (–38%), number of neutrophils (–48%), and MPO activity (–33%) were significantly lower in Fru+Hon-fed mice compared with Fru-fed mice (Figure 2A, and C–E). Also, Oil Red O staining of liver sections revealed lower lipid accumulation in livers of Fru+Hon-fed mice compared with Fru-fed mice (representative pictures are shown in Figure 2B). However, NAS and liver-to-body weight ratios as well as Oil Red O staining were still higher in livers of Fru+Hon-fed mice than in controls (Table 1 and Figure 2C). Fasting blood glucose levels did not differ between groups (Table 1). Moreover, hepatic protein levels of IL-6 were significantly higher in Fru-fed mice compared with all other groups whereas IL-6 protein levels in Fru+Hon-fed mice were still higher than in controls (Figure 2F). In line with these findings, the hepatic concentration of TBARS, a marker for lipid peroxidation [35], was significantly higher (+1.8-fold) in Fru-fed mice compared with Fru+Hon-fed mice (Table 1). PAI-1 protein levels in the livers of Fru- and Fru+Hon-fed mice were significantly higher compared with both control groups (Table 1).

**TABLE 1:** Effect of honokiol on body and liver weight, fasting blood glucose as well as liver damage in mice with fructose-induced MASLD.

	Diet and treatment groups			
	C	Fru	C + Hon	Fru + Hon
Body weight (g)	21.4 ± 0.3	22.7 ± 0.6	20.9 ± 0.5	22.4 ± 0.5
Liver weight (g)	1.0 ± 0.0	1.4 ± 0.0^1^^,^^2^	1.1 ± 0.0	1.4 ± 0.0^1^^,^^2^
Liver-to-body weight ratio (%)	5.0 ± 0.1	6.0 ± 0.1^1^^,^^2^	5.2 ± 0.1	6.0 ± 0.1^1^^,^^2^
Fasting blood glucose (mg/dL)	115.1 ± 3.64	120.7 ± 4.3	122.1 ± 5.5	122.8 ± 3.1
TBARS (μM/g protein)	61.1 ± 6.9	86.7 ± 12.4^3^	60.3 ± 6.7	47.4 ± 7.5
PAI-1 concentration (pg/mL per mg protein)	27.3 ± 1.5	91.5 ± 11.8^1^^,^^2^	31.2 ± 5.5	60.1 ± 9.1^1^^,^^2^

Data are shown as means ± SEM, *n* = 6–7.

Abbreviations: C, control diet; Fru, fructose; Hon, honokiol; MASLD, metabolic dysfunction-associated steatotic liver disease; PAI-1, plasminogen activator inhibitor-1; TBARS, thiobarbituric acid reactive substances.

1 *P* ≤ 0.05 compared with mice fed a control diet.

2 *P* ≤ 0.05 compared with mice fed a control diet and treated with 10 mg honokiol/kg bw.

3 *P* ≤ 0.05 compared with mice fed a 30% fructose solution and treated with 10 mg honokiol/kg bw.

### Effect of Hon on intestinal permeability and NO metabolism in mice with Fru-induced MASLD

**FIGURE 3:**
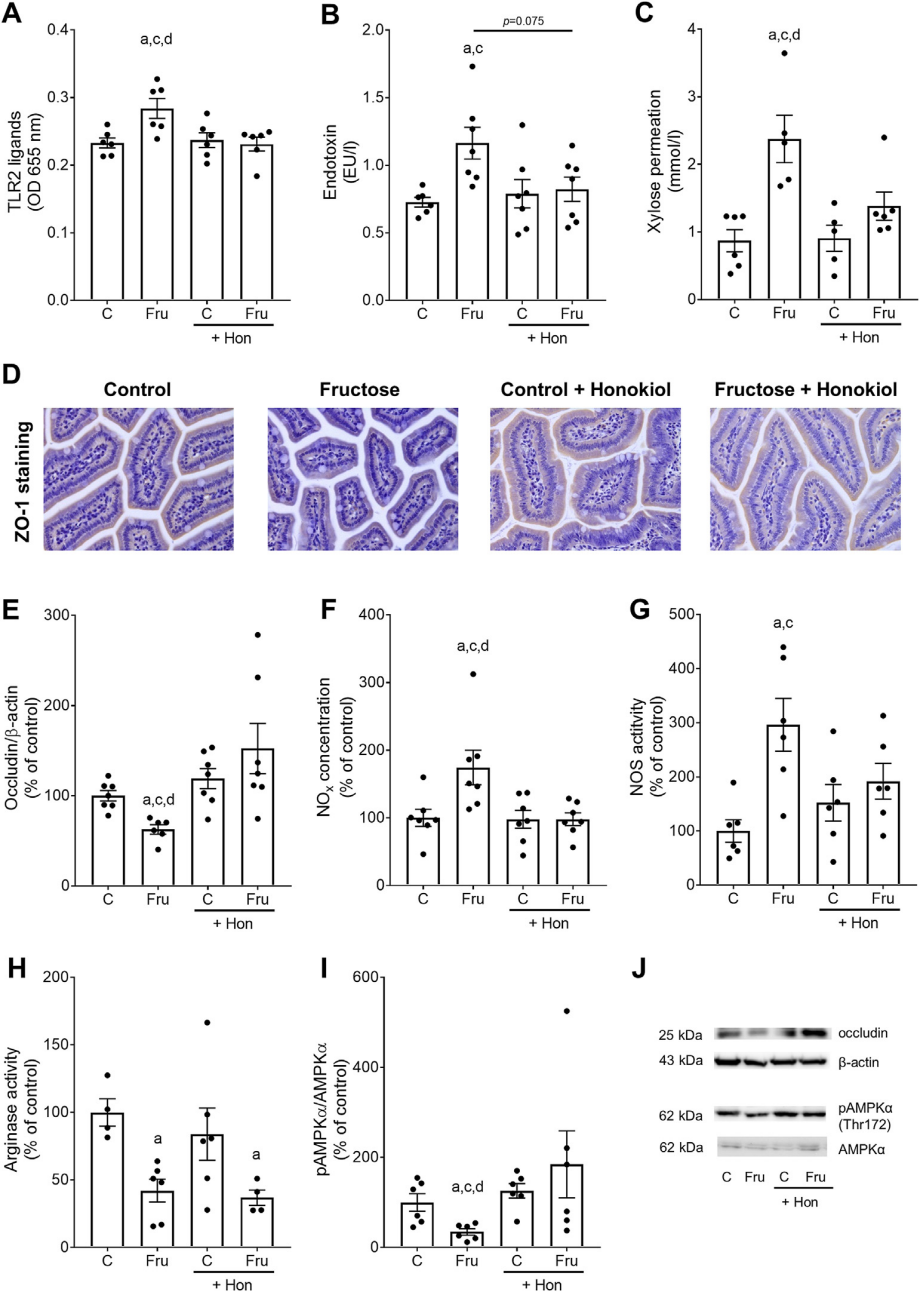
Effect of honokiol on intestinal permeability and markers of NO homeostasis in small intestine in mice with fructose-induced MASLD. (A) Concentration of Toll-like receptor 2 (TLR2) ligands, (B) bacterial endotoxin concentration in portal plasma, (C) permeation of xylose in small intestine, (D) representative pictures of zonula occludens (ZO-1) in small intestine (magnification 400×) as well as (E) occludin protein concentration. (F) Nitrite (NOx) concentration in small intestine, (G) nitric oxide synthase activity (NOS) and (F) arginase activity in murine enterocytes, (I) relative levels of phospho-5'AMP-activated protein kinase (pAMPKα) protein to AMPKα in small intestine and (J) representative blots of occludin, β-actin, pAMPKα, and AMPKα. Data are presented as means ± SEM, *n* = 5–7, except for (G) and (H): *n* = 4–6 as it was not possible to isolate enterocytes from each mouse in an appropriate amount for performing the assays. ^a^*P* ≤ 0.05 compared with mice fed a control diet, ^c^*P* ≤ 0.05 compared with mice fed a control diet and treated with 10 mg honokiol/kg bw, ^d^*P* ≤ 0.05 compared with mice fed a 30% fructose solution and treated with 10 mg honokiol/kg bw. AMPK, AMP-activated protein kinase; C, control diet; Fru, 30% fructose solution; Hon, honokiol; MASLD, metabolic dysfunction-associated steatotic liver disease; NO, nitric oxide.

The levels of TLR2 ligands and bacterial endotoxin in portal plasma of Fru-fed mice, being indicative of an intestinal barrier function, were significantly and by trend higher in Fru-fed mice than in all other groups (TLR2 ligands: Fru compared with Fru+Hon, +1.2-fold, *P* < 0.05; endotoxin: Fru compared with Fru+Hon, +1.4-fold, *P* = 0.075; all other comparisons *P* < 0.05). TLR2 ligand and bacterial endotoxin levels were at the level of controls in portal plasma of Fru+Hon-fed mice (Figure 3A and B). In line with these findings, the permeation of xylose as assessed in everted intestinal tissue sacs ex vivo at the time of killing was significantly higher in mice receiving 30% Fru solution (Figure 3C) than in all other groups, while being at the level of controls in Fru+Hon-fed mice. Protein levels of ZO-1 were lower in the small intestine of Fru-fed mice compared with C-, C+Hon-, and Fru+Hon-fed mice, but did not differ between the latter groups. Representative pictures of staining are shown in Figure 3D. In line with these findings, protein levels of occludin were significantly lower in Fru-fed mice compared with all other groups, whereas occludin protein levels in small intestinal tissues of Fru+Hon-fed mice were almost at the level of control (Figure 3E and J).

As recent publications have linked Fru-induced intestinal barrier dysfunction to alterations in intestinal nitric oxide (NO) homeostasis (for overview, see [4]), we next determined markers of NO metabolism. Indeed, NOx levels in the small intestine of Fru-fed mice were significantly higher than in all other groups, while being at the level of controls in Fru+Hon-fed mice (Figure 3F). NOS activity was significantly higher in enterocytes of Fru-fed mice than in control groups. Similar differences were not found when assessing NOS activity in enterocytes isolated from Fru+Hon-fed mice (Figure 3G). In line with our previous findings [22, 32], arginase activity, the counterpart of inducible NOS (iNOS) and also being critical for maintaining NO homeostasis [37], was significantly lower (–2.4-fold) in enterocytes of Fru-fed mice compared with C-fed mice (Figure 3H). However, the loss of arginase activity found in Fru-fed mice was not affected by Hon. Moreover, the ratio of Thr172 phosphorylated AMPKα (pAMPKα) to total AMPKα, being indicative of maximal AMPK activity on the cellular level [38,39], was significantly lower in the small intestine of Fru-fed mice compared with all other groups whereas the ratio was at the level of controls in Fru+Hon-fed mice (Figure 3I, J and Supplemental Figure 1).

### Effect of Hon on markers of intestinal permeability and markers of NO metabolism in everted gut sacs of mice and Caco-2 cells

**FIGURE 4:**
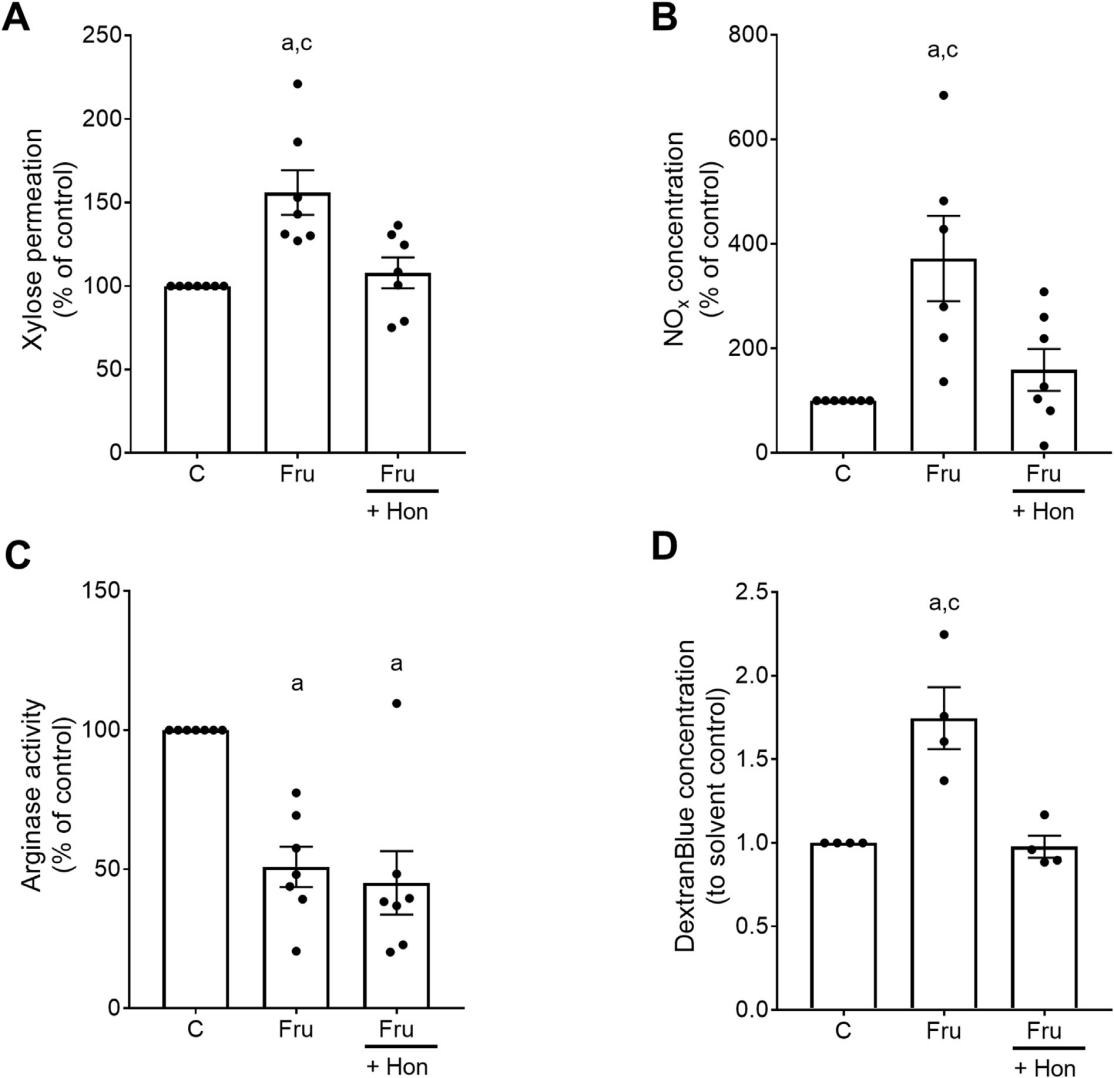
Effect of honokiol on intestinal permeability, markers of NO homeostasis in everted gut sacs of naïve female C57BL/6J mice and Caco-2 cell experiments. (A) Permeation of xylose, (B) concentration of nitrite (NOx) levels, (C) arginase activity in small intestine of everted gut sacs as well as (D) DextranBlue concentration in the basolateral compartment of the Caco-2 cell monolayer. Data are presented as means ± SEM, for the ex vivo everted sac experiments: *n* = 7–8, ^a^*P* ≤ 0.05 compared with everted gut sacs incubated in 1× KRH buffer, ^c^*P* ≤ 0.05 compared with everted gut sacs incubated in 5 mM Fru + 1 μM honokiol solution, for the Caco-2 cell experiments: *n* = 4, ^a^*P* ≤ 0.05 compared with untreated Caco-2 cells, ^c^*P* ≤ 0.05 compared with Caco-2 cells treated with 5 mM Fru + 1 μM honokiol. C, untreated everted gut sacs or cells; Fru, 5 mM fructose; Hon, honokiol.

To further assess whether the molecular mechanism underlying the protective effects of Hon on Fru-induced intestinal barrier dysfunction was due to a direct effect of Hon on the small intestine and enterocytes, respectively, we next performed ex vivo everted gut sac experiments in which everted small intestinal tissue sacs were treated with 5 mM Fru ± 1 μM Hon (see also experimental setup in Figure 1A). In line with the in vivo findings, intestinal permeability, i.e. xylose permeation, was significantly higher in Fru-treated everted gut sacs (+1.6-fold) compared with everted gut sacs incubated in KRH buffer alone (Figure 4A). These effects were almost attenuated when Fru-challenged everted gut sacs were concomitantly treated with Hon (Figure 4A). The concomitant treatment of everted gut sacs with Hon significantly attenuated the Fru-induced increase of NOx concentration (Figure 4B) whereas Hon had no effect on the Fru-associated decrease in intestinal arginase activity (Figure 4C). Moreover, the increased permeation of DextranBlue found when differentiated Caco-2 cells were exposed to Fru was significantly attenuated (–44%) when cells were concomitantly treated with Hon (experimental setup and results are shown in FIGURE 1, FIGURE 4D).

## Discussion

With a prevalence of ∼32%, MASLD is the leading cause of liver disease worldwide [2]. Several epidemiological and clinical studies suggest that besides genetic predisposition, age, and gender, nutritional factors including overnutrition and a diet rich in saturated fats and sugars, particularly a high consumption of Fru, are discussed to be critical to develop insulin resistance and metabolic liver diseases ([40,41] and for overview see [42]). In line with the findings in previous studies of our group [9,43] and others [44], the intake of a 30% Fru solution led only to early signs of MASLD, e.g. hepatic steatosis with beginning inflammation. This was evaluated using the semiquantitative “Nonalcoholic Steatohepatitis Clinical Research Network System for Scoring Activity and Fibrosis in Nonalcoholic Fatty Liver Disease” modified from Kleiner et al. [27] and Brunt [28]. Also, in line with previous studies, mice fed a 30% Fru solution showed no histological signs of fibrosis. In contrast, the addition of Hon to the Fru-enriched drinking water diminished the development of fatty liver and inflammatory processes, such as the increase in the number of neutrophils and MPO activity, as well as of PAI-1 and IL-6 protein concentration in liver tissue. In contrast, although having been identified as one of the most critical risk factors in the development of MASLD, fasting glucose levels were not altered by Fru ingestion regardless of additional treatments. This is in line with previous findings of our group employing the same feeding model for even an extended period, e.g. 8 wk, revealing only limited effects on markers of insulin resistance [21]. Indeed, while the feeding model used in this study leads to the development of early signs of MASLD (fat accumulation in the liver and an increase in some inflammatory markers), further studies, employing either a longer feeding period or a combination of a Fru- and fat-rich diet, are needed to determine whether Hon also affects the development of later stages of MASLD like MASH or fibrosis.

Our findings regarding the effect of Hon at lower doses in this study are in line with those of others using higher or even pharmacological doses of Hon (20–100 mg Hon/kg bw/d) also reporting a protective effect of the neolignan in the setting of high-fat diet- and MCD-induced MASLD, respectively [18]. In conclusion, the results of this study suggest that even low doses of Hon may attenuate not only hepatic fat accumulation but also inflammatory alterations including the recruitment of neutrophils. However, whether the dose of Hon used in this study also affects the development of the later stages of MASLD or when Fru is combined with other macronutrients like fat, remains to be determined.

### Hon attenuated Fru-induced intestinal barrier dysfunction

Several studies suggest that besides its insulin-independent metabolism, Fru may contribute to the development of MASLD by impairing intestinal barrier function (for overview, see [45]). This in turn has been shown to lead to increased translocation of bacterial endotoxin but also other pathogen-associated molecular patterns. Studies have shown that bacterial endotoxin adds to the development and progression of MASLD in both humans and rodents by activating TLR4-depending signaling cascades in the liver [43,46,47]. Studies in rodents suggest that the chronic intake of Fru is associated with a loss of tight junction proteins in the small intestine, translocation of bacterial toxins, and subsequent induction of the activation of the TLR4 but also other TLR signaling cascades [43,48]. However, the underlying molecular mechanisms of Fru-induced MASLD are still not fully understood. In this study, the protective effects of an oral supplementation of Hon were associated with a protection against the increase in small intestinal permeability as determined by the permeation of xylose, lower levels of bacterial toxins in portal plasma, and lower protein levels of the tight junction proteins ZO-1 and occludin. In line with these in vivo findings, Hon also attenuated the Fru-dependent increase in permeability in small intestinal everted tissue sacs as well as in differentiated Caco-2 cells used as a model of human small intestinal cells. Results of animal studies administrating Hon i.p. suggest that the neolignan may alleviate gastrointestinal dysfunction in the setting of an enterotoxigenic *Escherichia coli*-induced diarrhea in mice, indicating that Hon may contribute to maintaining intestinal mucosal integrity and homeostasis [49]. Somewhat in line with the findings of this study, the results of Wang et al. [19] suggest that oral treatment with higher doses of Hon (40 mg/kg wt) alleviated DSS-induced colitis in C57BL/6 mice. The protective effects of Hon were related to a reduction in markers of oxidative stress and inflammatory processes as well as improving colonic intestinal barrier function by “normalizing” the mRNA expression and protein levels of the tight junction proteins ZO-1, occludin, and claudin 1 [19].

Recent studies from our group suggest that intestinal barrier dysfunction in settings of diet-induced MASLD is associated with alterations in NO homeostasis, including an increased formation of NO and a loss of arginase activity in the small intestine [22,32], and that targeting these alterations may attenuate the development of intestinal barrier dysfunction and subsequently the development of MASLD [22,32]. In this study, the protective effects of the supplementation of Hon were associated with an attenuation of the increase of NOx and NOS activity found in mice only fed Fru. Moreover, although not altering the decrease in arginase activity, the loss of phosphorylation of AMPK being indicative of AMPK activity, was attenuated in small intestinal tissue of Fru-fed mice concomitantly treated with Hon. AMPK has been shown to be critical in the regulation of both iNOS and intestinal barrier function [50]. Moreover, Wang et al. [19] also reported that in vitro using RAW264.7 cells as a model, the protective effect of Hon on inflammatory processes including the induction of iNOS was related to activation of AMPK. The underlying molecular mechanisms remain to be determined. The activity of arginase, proposed to be the counterplayer of (i)NOS [37], being lower in tissues and mice exposed to Fru, was unaffected by the treatment with Hon. Indeed, NOS and arginase can both antagonize or synergize in the generation of oxidative and nitrosative stress [51]. An imbalance of arginase and NOS activity is discussed to be a trigger of intestinal barrier dysfunction (for overview, see [4]). It could be that the attenuation of the increased formation of NOx and NOS activity found in enterocytes of Fru-fed mice treated with Hon was sufficient to “rescue” this imbalance. Whether this is related to a regulatory effect of Hon on NOS activity at (post)transcriptional and/or (post)translational level remains to be determined. Moreover, although not showing a marked effect on intestinal microbiota in mice fed Hon [18], it could be that changes in the metabolism of intestinal microbiota could have added to the protective effects found in mice in this study. This needs to be determined in future studies. Furthermore, it remains to be determined whether an oral supplementation of Hon also affects the progression of MASLD to later stages of the disease and whether these effects are also found in patients with MASLD.

In conclusion, the results of this study suggest that an oral supplementation of Hon may attenuate the development of Fru-induced MASLD and that this is related to protection against the development of intestinal barrier dysfunction in the small intestine. Moreover, our results further bolster the hypothesis that Hon may alter AMPK and NOS activity even when consumed at lower doses.

## Author contributions

The authors’ responsibilities were as follows – VMD, IB: conceptualization, funding, supervision; ABaumann: visualization; ABaumann, IB: writing original draft preparation; and all authors: data curation or formal analysis, investigation, writing – review and editing, read and agreed to the final version of the manuscript.

## Data availability

Data are available from the corresponding author on reasonable request.

## Funding

The study was in part funded by the Interdisciplinary Network of the Faculty of Life Sciences, University of Vienna to VMD and IB. Open Access funding provided by University of Vienna.

## Conflict of interest

Ina Bergheim is an Editorial Board Member for *The Journal of Nutrition* and played no role in the Journal's evaluation of the manuscript. All other authors report no conflicts of interest.
