# Supplementary material for: Honokiol, a Neolignan from Magnolia officinalis, Attenuated Fructose-Induced Hepatic Fat Accumulation by Improving Intestinal Barrier Function in Mice
Source: J Nutr. 2025 Feb 21;155(4):1173–82. doi: 10.1016/j.tjnut.2025.02.017 (PMC12107254; doi:10.1016/j.tjnut.2025.02.017)
Supplement: multimedia component 1 [file mmc1.pdf]

Honokiol, a neolignan from *Magnolia officinalis*, attenuated fructose-induced hepatic fat accumulation by improving intestinal barrier function in mice

Baumann et al.

Supplemental Table S1. Nutrient composition of diet (V1534-300, pellets, fortified, Ssniff, Soest, Germany).

Crude protein (N × 6.25)	19.0%
Crude fat	3.3%
Crude fibre	4.9%
Crude ash	6.4%
Starch	35.2%
Sugar	5.3%
N free extracts	54.2%
Lysine	1.10%
Methionine	0.38%
Cystine	0.35%
L-Met + L-Cys	0.73%
L-Threonine	0.72%
Tryptophan	0.25%
Arginine	1.19%
Histidine	0.49%
Valine	0.92%
Isoleucine	0.79%
Leucine	1.39%
Phenylalanine	0.88%
Phe+Tyr	1.50%
Glycin	0.88%
Glutamic acid	4.22%
Aspartic acid	1.84%
Proline	1.31%
Serine	1.01%
Alanine	0.87%
Calcium	1.00%
Phosphorus	0.70%
Sodium	0.24%
Magnesium	0.22%
Potassium	0.92%

Vitamin A (retinol acetate)	15,000 IU/kg
Vitamin D_3 (cholecalciferole)	1,500 IU/kg
Vitamin E (α-tocopherol acetate)	135 mg/kg
Vitamin K	20 mg/kg
Vitamin B1	86 mg/kg
Vitamin B2	32 mg/kg
Vitamin B6	31 mg/kg
Vitamin B12	100 µg/kg
Nicotinic acid	150 mg/kg
Pantothenic acid	59 mg/kg
Folic acid	10 mg/kg
Biotin	710 µg/kg
Choline	1,370 mg/kg
Iron	186 mg/kg
Manganese	68 mg/kg
Zinc	91 mg/kg
Copper	15 mg/kg
Iodine	2.1 mg/kg
Selenium	0.3 mg/kg
Fatty acids	
12:0	-
14:0	0.01%
16:0	0.45%
18:0	0.09%
20:0	0.01%
18:1 (n-9)	0.62%
18:2 (n-6)	1.76%
18:3 (n-3)	0.23%
Metabolizable energy (ME)	13.5 MJ/kg
CP	24 kJ%
CL	9 kJ%
Carbohydrate, CHO	67 kJ%

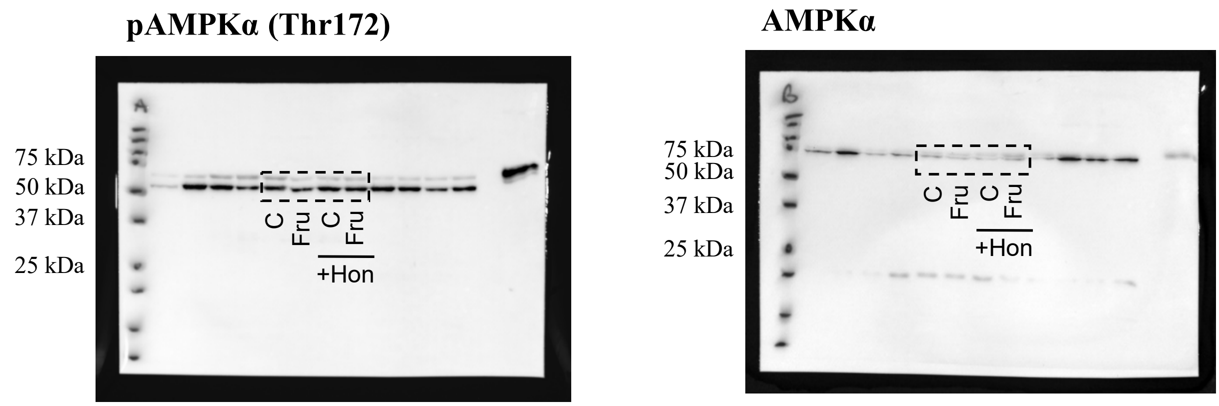

Supplemental Figure S1: Pictures of whole Western Blot of pAMPKα and AMPKα in small intestine in mice fed a 30% fructose solution supplemented with honokiol. Bands that are shown at high magnification in Figure 3J are highlighted with a frame. AMPK: 5´AMP-activated protein kinase; C: control diet; Fru, 30% fructose solution; Hon, honokiol.
